# A Hospital Chairman's Grievances
*The first article appeared in The Hospital of Feb. 24, page 542.


**Published:** 1912-03-09

**Authors:** 


### A HOSPITAL CHAIRMAN'S GRIEVANCES.*

### II.—Complaints and their Investigation.

As I turn back over my files of correspondence many curious memories awaken which I must not elaborate too fully, as many individuals concerned would perhaps have their feelings hurt.

I remember once a case where the only complaint was that no medicine had been given. It was a surgical case that came for dressing. The case, unfortunately, died shortly after the last attendance. In another a medical man was alleged to have said that the hospital surgeons were " solely responsible " for the death. I ascertained his name and address, went round myself to his surgery, and was shown into the waiting-room. He was a sixpenny doctor. When I arrived there were some fifteen patients waiting, and before I left there were about thirty. If the time he took over each case was a gauge of his care I can testify thereto, as, although I arrived about 8 p.m., I calculated that it would be nearly 10 before my turn came, and could not surmise when he would be able to conclude for the day. The conversations to which I listened as I pretended to read my evening paper could only be done justice to by a Dickens, or perhaps at the present date, a Pett Ridge.

At last I decided to leave for that evening, and write for an appointment at an early date. The waiting-room was just at the top of a short flight of stairs and the consulting-room at the bottom. A small boy acted as page, and was waiting outside the latter room to speed the parting patient and usher in the next. As I reached the foot of the stairs a patient came out. I told the boy I would write for an appointment, but he insisted on having my name and business. While we were talking most of the waiting patients crowded to the top of the stairs, and their audible remarks were far from complimentary. I feel sure I should have suffered damage if some of them could have had their wish, as they naturally thought that I too was a patient, perhaps better able to afford to go to a less busy man than they; and they naturally resented my stealing a march on them, as they thought. Meanwhile the doctor came out, took my card from the page, and insisted on my going in. He remembered the case well, and stated that far from blaming the hospital people, he blamed the parents, who could well have afforded to call in a doctor, for taking the case to the hospital in inclement weather instead of sending for a medical man. He was good enough to confirm this in writing.

I have his letter now, which concluded with an unsolicited testimonial to the care and attention that any patients he had sent to the hospital had invariably received. As I left I asked the obliging boy to apologise to the outraged waiters and to say that I was not a patient, but had come about an urgent case. I glanced up as I gave the message, and my last impressions confirmed my first, that it was time to go. The house surgeon afterwards told me that medicine is frequently not given in surgical cases, and that some patients cannot understand that it may be unnecessary. Further, that he had often to expostulate with parents for bringing children to the hospital when they were only fit to be kept as warm as possible. The above case is typical of a large number of investigations which I have made in which there has been no solid justification for complaint. Chairman.

* The first article appeared in The Hospital of Feb. 24, page 542.

